# Augmented myeloablative conditioning with olaparib in allogeneic hematopoietic stem cell transplantation for relapsed/refractory RUNX1::RUNX1T1-positive acute myeloid leukemia

**DOI:** 10.3389/fonc.2026.1608899

**Published:** 2026-03-25

**Authors:** Song Xue, Wei Ma, Man Chen, Hui-peng Sun, Xing-yu Cao

**Affiliations:** 1 Department of Bone Marrow Transplant, Beijing Lu Daopei Hospital, Beijing, China; 2 Department of Bone Marrow Transplant, Hebei Yanda Lu Daopei Hospital, Langfang, China; 3 Division of Pathology & Laboratory Medicine, Beijing Lu Daopei Hospital, Beijing, China

**Keywords:** allogeneic hematopoietic stem cell transplantation, conditioning regimen, olaparib, PARP inhibitor, relapse, RUNX1::RUNX1T1

## Abstract

**Background:**

Relapsed/refractory RUNX1::RUNX1T1-positive acute myeloid leukemia remains a therapeutic challenge due to high relapse rates post-allogeneic hematopoietic stem cell transplantation. Preclinical evidence suggests that RUNX1::RUNX1T1 fusion proteins sensitize cells to poly ADP-ribose polymerase inhibitors by suppressing homologous recombination repair. This study explores the safety and efficacy of a novel conditioning regimen incorporating olaparib (a PARPi) for relapsed/refractory RUNX1::RUNX1T1+ AML patients undergoing allo-HSCT.

**Methods:**

A retrospective analysis was conducted on six relapsed/refractory RUNX1::RUNX1T1+ AML patients treated between June 2022 and October 2023 at Beijing Lu Daopei Hospital. The conditioning regimen included olaparib, FLAG, busulfan, and cyclophosphamide. Outcomes were evaluated for engraftment, toxicity, molecular remission, and survival.

**Results:**

All six patients achieved complete molecular remission within three months post transplantation. Neutrophil and platelet engraftment occurred at a median of 15 days each. Acute graft-versus-host disease occurred in four patients, and chronic GVHD was observed in all cases. Viral reactivation (CMV/EBV) occurred in four patients. At a median follow-up of 25 months, all patients remained leukemia-free, with a 100% leukemia-free survival rate. No toxicity-related deaths or unexpected adverse events were reported.

**Conclusion:**

The incorporation of olaparib into the conditioning regimen demonstrated favorable safety and efficacy, inducing rapid CMR and durable remission in high-risk relapsed/refractory RUNX1::RUNX1T1+ AML patients. These findings highlight the potential of PARPi-enhanced conditioning to overcome chemotherapy resistance and reduce relapse by targeting leukemia stem cells (LSCs) through immune-mediated mechanisms. Small-sample retrospective studies have inherent limitations; hence further prospective studies are warranted to validate these results.

## Introduction

Acute myeloid leukemia (AML) is a clinically and genetically heterogeneous disease, AML with the t(8;21) (q22;q22) chromosomal translocation, resulting in the formation of fusion proteins, is generally associated with a favorable outcome, but AML with positive has shown to have clinical heterogeneity and relapse occurs in about 30% of patients (1, 2). In fact, RUNX1::RUNX1T1 transcript levels, when measured post-treatment and coupled with the KIT mutation status, constitute a potent marker to predict potential relapse (2).High-risk RUNX1::RUNX1T1 AML patients benefited from allo-HSCT (allogeneic hematopoietic stem cell transplantation).Despite undergoing allo-HSCT in the optimal first complete remission(CR1) status, the relapse rate among this cohort of patients remained at 22.1%-38.4% (2, 3). In transplants performed during the second or later CR, or during active disease, the relapse rate tends to be significantly elevated. In the initial three months post allo-HSCT, an inadequate RUNX1::RUNX1T1 transcript levels decline indicates a heightened risk of disease recurrence (4). Adequate pre-conditioning regimen administered prior to transplantation are crucial for eliminating leukemia cells and achieving optimal gene decline levels. Recent research indicates that the activity of DNA damage response (DDR)-related transcription factors can be modulated by RUNX1::RUNX1T1 fusion oncoprotein. Notably, the RUNX1::RUNX1T1 has been identified to sensitize cells to poly ADP-ribose polymerase inhibitors (PARPi), this effect is attributed to the suppression of crucial genes involved in homologous recombination repair (HRR) mediated by RUNX1::RUNX1T1 (5). Therefore, incorporating PARPi-olaparib into the conditioning regimen for relapsed/refractory RUNX1::RUNX1T1+ AML has the potential to augment the effectiveness of transplantation. Herein, we report the first case series of allo-HSCT utilizing a conditioning regimen incorporating olaparib for relapsed/refractory RUNX1::RUNX1T1+ AML patients.

## Patients and methods

A retrospective analysis was conducted at Beijing Lu Daopei Hospital to evaluate the effectiveness of allo-HSCT, preceded by an olaparib containing novel conditioning regimen, in treating relapsed/refractory RUNX1::RUNX1T1+ AML patients. Data was prospectively collected from medical record database. This study was approved by the Biomedical Ethics Committee of Lu Daopei Hospital (DPEC-202511).

### Patients

Consecutive relapsed/refractory RUNX1::RUNX1T1+ AML patients who underwent allo-HSCT utilizing the novel conditioning regimen were enrolled at Beijing Lu Daopei Hospital between June 2022 and October 2023. The following inclusion criteria were applied: (1) Age14 to 60 years old; (2)≥CR2 or active disease; (3) no contraindications to allo-HSCT; (4) first allo-HSCT; (5) patients who signed informed consent forms. RUNX1::RUNX1T1+ AML with systemic mastocytosis was excluded from the study. Written informed consent was obtained from all patients or guardians before entry into the study in accordance with the Declaration of Helsinki.

### Treatment protocols

The conditioning regimen used in the patients consisted of olaparib, FLAG, busulfan and cyclophosphamide. The detailed process is as follows: olaparib 150mg bid from days -15 to -4; granulocyte colony-stimulating factor (G-CSF) 5μg/kg/d from days -14 to -10; fludarabine 30mg/m^2^/d from days -14 to -10; Ara-C (cytarabine) 2g/m^2^/d from days -14 to -10; busulfan(3.2 mg/kg/d) from days -9 to -6; Cyclophosphamide (1.0g/m^2^/d) from days -5 to -4; Tacrolimus(the initial dose of tacrolimus was 0.015 mg/kg/day and was started on days -1 and continued intravenous infusion for 24h.The dose of tacrolimus was adjusted according to the drug blood concentration, the blood concentration of tacrolimus was maintained within the range of 5-8 ng/ml.), mycophenolate mofetil, intravenous methotrexate(15 mg/m^2^ on days +1, then 10 mg/m^2^ on days+3 and days+6) were used for the prophylaxis of graft-versus-host (GVHD) for matched-related allo-HSCT, this protocols combined with anti-human thymocyte immunoglobulin (1.875 mg/kg/d, Genzyme, from days -5 to -2) were used for the prophylaxis of GVHD for haploidentical allo-HSCT and matched-unrelated allo-HSCT.

### Disease monitoring

As part of the treatment protocol, bone marrow (BM) samples were collected before transplantation and serially thereafter at 1, 2, 3, 6, 9, 12, 15, 18, 21, 24, 30, 36, 42, 48, 54 and 60 months post allo-HSCT. MRD was monitored using RQ-PCR to quantify the level of RUNX1::RUNX1T1 transcripts according to recommendations of the Europe Against Cancer Program. The RUNX1-RUNX1T1 transcript level was calculated as the percentage of RUNX1-RUNX1T1 transcript copies/ABL1 copies (ABL1>10^4^). In patients with extramedullary lesions, PET-CT imaging was routinely employed for the assessment of the lesions.

### Definitions

CR was defined as bone marrow blasts <5%, absence of circulating blasts and extramedullary disease, MRD-negative was defined as CR with negativity by multiparameter flow cytometry (FCM). Major molecular remission (MMR) was defined as 3-log reduction in RUNX1::RUNX1T1 transcripts when compared with the baseline before allo-HSCT (6). Complete molecular remission (CMR) was defined as a reduction in RUNX1::RUNX1T1 transcript levels to 0. Acute GVHD (aGVHD) was diagnosed and graded using previously published consensus criteria (7), Chronic GVHD (cGVHD) was defined and graded according to the National Institutes of Health consensus criteria (8). Overall survival (OS) was defined as the time from the first day of transplantation to death due to any cause or the last follow-up. Leukemia-free survival (LFS) was defined as the time from transplantation to relapse or death, or last follow-up. Relapse was defined as the reappearance of blasts in blood, bone marrow (>5%) or any extramedullary site after achieving CR or loss MRD-negative. Non-relapse mortality (NRM) was defined as death without relapse.

## Results

### Patient characteristics

A total of six relapsed/refractory RUNX1::RUNX1T1+ AML patients were enrolled in this study, the male: female ratio was 1:1 and the median age was 36 (range, 14–59) years. Of the six patients, four presented with extramedullary lesions, one of whom concurrently exhibited of the central nervous system leukemia. All six patients presented with one or more gene mutations, primarily KIT and FLT3, while BCORL1, CSF3R, TET2, ASXL, and BRCA2 mutations were also observed. Among the six patients, two achieved complete remission following one cycle chemotherapy, whereas the remaining four attained complete remission after two cycles. Following a median remission duration of 14.5 (range, 10-15) months, all six patients experienced relapse after achieving complete remission. Of these patients, three attained re-remission after salvage therapy, whereas the remaining three continued to exhibit an active disease state. More details are listed in **Table 1**.

**Table 1:** Patients characteristic pre-HSCT in the study.

UPN	Age	Gender	Diagnosis time	WBC	EM lesions	CNSL	Gene mutation	Course acquired to achieve CR1	Relapse time	AML1::ETO levels pre-HSCT	EM lesions pre-HSCT	Disease status pre-HSCT
1	26	Female	2021.3.	7.05	Yes	No	BCORL1	1	2022.5	0.51%	No	CR2
2	46	Male	2021.5	64.6	Yes	Yes	FLT3-TKD,CSF3R	2	2022.8	133.43%	Yes	AD
3	14	Female	2021.12	11	Yes	No	KIT(D816Y,D816V)	2	2022.10	0.1%	Yes	AD
4	47	Female	2022.8	13.74	No	No	KIT(D816V), FLT3-TKD	1	2023.12	0.15%	No	CR2
5	15	Male	2022.1	22.67	Yes	No	FLT3-ITD,CSF3R, TET2	2	2023.3	0.11%	Yes	AD
6	59	Male	2022.9	12.8	No	No	KIT(D642E),ASXL, BRCA2	2	2023.7	0.8%	No	CR2

UPN, unique patient number; WBC, white blood cell count; EM, extramedullary; CNSL, central nervous system leukemia; CR, complete remission; HSCT, hematopoietic stem cell transplantation; AD, active disease.

### Patient outcomes

All six patients tolerated the conditioning regimen well. Diarrhea, mucositis, and neutropenic fever were the most frequently observed toxicity reactions. Of the six patients, five underwent haploidentical allo-HSCT, while one underwent a HLA matched sibling allo-HSCT, the median infused mononuclear cell (MNC) count was 10.625 × 10^8^/kg (range: 8.07-13.57), while the median infused CD34+ cell count was 5.985 × 10^6^/kg (range: 5-7.37).Patients who underwent haploidentical allo-HSCT received an infusion of unrelated cord blood (UCB) from a third-party donor (HLA matching requirement was 3/6 using low-resolution HLA typing for HLA-A, -B and-DRB1) at a unified cell dose of 1.0×10^7^/kg total nucleated cells (TNCs). All patients successfully underwent hematopoietic reconstitution, with a median neutrophil engraftment time of 15 days (range: 11-17) and a median platelet engraftment time of 15 days (range: 11-21). Among the six patients, four exhibited acute graft-versus-host disease (aGVHD) of varying severity, with one case of grade 1 (UPN3,unique patient number3), two cases of grade 2 (UPN2, UPN5), and one case of grade 3 (UPN1).All cases of aGVHD were effectively controlled following treatment with glucocorticoids and/or CD25 monoclonal antibody. All six patients displayed manifestations of chronic graft-versus-host disease (cGVHD), with five cases showing mild symptoms and one case (UPN4) demonstrating moderate severity. All mild cGVHD cases were effectively controlled after adjusting the dosage of tacrolimus and administering glucocorticoid therapy. However, UPN4 developed capillary leak syndrome as a complication of chronic GVHD, which improved following treatment with bevacizumab (9). In the cohort of six patients, three cases (UPN3, UPN4, UPN6) of cytomegalovirus viremia and one case (UPN4) of Epstein-Barr virus viremia were observed. All cases of cytomegalovirus viremia became negative after pre-emptive therapy; additionally, the Epstein-Barr virus viremia in UPN4 resolved following a reduction of immunosuppressive intensity. After one month of transplantation, two patients (UPN4, UPN5) displayed persistent RUNX1::RUNX1T1 transcript levels in bone marrow. In the second month following transplantation, one patient (UPN4) continued to exhibit a detectable RUNX1::RUNX1T1 transcript level. In the third month post-transplantation, all six patients exhibited 0 RUNX1::RUNX1T1 transcript levels. All six patients exhibited CMR responses within three months following transplantation. PET-CT assessment demonstrated that patients with extramedullary lesions achieved remission. By March 30, 2025, All patients who achieved CMR have maintained this status continuously, demonstrating a median OS and LFS of 25 months (range: 18-32) post-transplantation. More details are listed in **Table 2**.

**Table 2:** HSCT-related information of patients in the study.

UPN	Donor resource	HLA matching	D/R sex match	D/R blood group match	Graft type	Transplant date	MNC (×108/kg)	CD34+Cells (×106/kg)	Time for implantation of neutrophils	Time for implantation of PLT	aGVHD	cGVHD	CMV activation	EBV activation	AML1::ETO Levels (1m)	AML1::ETO Levels (2m)	AML1::ETO Levels (3m)
1	HID	5/10	F/F	A/A	BM+PB+UCB	2022.7.7-8	8.07	5	15	17	grade III (gut stage 2)	mild	no	no	0	0	0
2	HID	6/10	M/M	AB/B	BM+PB+UCB	2022.9.21-22	8.28	5.05	15	15	grade II (gut stage 1)	mild	no	no	0	0	0
3	HID	5/10	F/F	AB/AB	BM+PB+UCB	2023.1.26-27	12.71	7.37	15	21	grade I (skin stage 1)	mild	yes	no	0	0	0
4	MSD	10/10	F/M	AB/B	BM+PB	2023.3.9-10	10.5	5.97	11	12	–	moderate	yes	yes	0.019%	0.006%	0
5	HID	5/10	M/M	B/B	BM+PB+UCB	2023.4.23-24	13.57	7	17	11	grade II (skin stage 3)	mild	no	no	0.08%	0	0
6	HID	5/10	F/F	B/B	BM+PB+UCB	2023.8.31-9.1	10.75	6	12	15	–	mild	yes	no	0	0	0

D, donor; R, recipient; F, Female; MNC, mononuclear cells; aGVHD, acute graft-versus-host disease; cGVHD, chronic graft-versus-host disease; HID, haploidentical donor; BM, bone marrow; PB, peripheral blood; UCB, unrelated cord blood; MSD, HLA matched sibling donor; M, Male.

## Discussion

PARP1 functions as a DNA damage sensor, detecting and binding to single-strand breaks (SSBs) to initiate the DNA repair response. Once the DNA is repaired, PARP1 disengages from the DNA, reverting to its catalytically inactive state. PARPi induces the formation of double-strand breaks (DSBs) through the inhibition of PARP enzyme activity or PARP trapping mechanisms. In cells with competent homologous recombination repair (HRR), DSBs can be repaired via HRR, resulting in cell survival (10). HRR is a BRCA1- or BRCA2-dependent mechanism utilizing a homologous DNA sequence as a template for repairing DSBs. Cells with BRCA1 or BRCA2 mutations exhibit defects in HRR, rendering replication forks obstructed by PARPi irreparable and often resulting in synthetic lethality in tumor cells (11). Tumors exhibiting ‘BRCAness’ or molecular features similar to BRCA-mutant tumors may also respond positively to these therapeutic strategies (12–14). RUNX1::RUNX1T1 fusion oncoproteins sensitize cells to PARPi by suppressing key HR-associated genes (5). In preclinical studies, acute myeloid leukemia harboring the following co-occurring mutations demonstrated high sensitivity to PARP inhibitors: signaling pathway gene mutations (FLT3-ITD combined with TET2 deficiency, or FLT3-ITD concurrently with TET2 and DNMT3A deficiency), cohesin complex members (STAG2), TP53 and BCOR as co-occurring lesions, and IDH1/2 mutations. These findings may help identify patient populations suitable for PARPi therapy (15). Our previous study showed that the combination of olaparib and chemotherapy was effective and safe in elderly patients with relapsed RUNX1::RUNX1T1+ AML (16). Additionally, *in vitro* studies demonstrated a promising synergistic effect between PARPi and busulfan (17). According to the aforementioned theory and evidence, incorporating olaparib into the conditioning regimen for allo-HSCT could potentially enhance RUNX1::RUNX1T1+ leukemic cell eradication efficacy, ultimately leading to a reduction in leukemia relapse, especially in patients with relapsed and refractory RUNX1::RUNX1T1+ AML patients.

Although this conditioning regimen was intensified in terms of dosage, patients exhibited good tolerance with no unexpected adverse effects or toxicity-related deaths. All patients successfully underwent engraftment of leukocytes and platelets, the incorporation of olaparib had no adverse impact on stem cell engraftment. Following transplantation, there was no notable rise in major complications, such as acute and chronic GVHD or viral reactivation. These results indicate a favorable safety profile for the novel protocol. Despite not undergoing allo-HSCT during the optimal CR1 phase, with half of the patients in active disease, all achieved CMR within three months post-transplantation. This favorable early treatment response ultimately resulted in long-term survival benefits. Currently, all patients are in a state of DFS. This indicates that early CMR post-transplantation is predictive of patients’ long-term prognosis. Additionally, the new conditioning regimen containing olaparib can induce early CMR. Consequently, this regimen improves the prognosis for this patient group.

The presence of leukemic stem cells (LSCs) is a key factor contributing to leukemia relapse post-treatment (18),and their eradication is a vital strategy to minimize post-transplantation relapse. LSCs commonly display resistance to chemotherapy and immune evasion, presenting substantial obstacles to their eradication (19). The DNA damage response can alert the immune system by upregulating NKG2D ligands (NKG2DL) to mark potentially dangerous cells, thereby facilitating their recognition and clearance by immune cells such as NK cells and T cells (20). The absence of surface expression of NKG2DL distinguishes LSCs from their more differentiated leukemic cells, enabling their immune evasion (21). A previous study demonstrated that PARP inhibition, induced the expression of NKG2DL in LSCs. These ligands subsequently bind to NKG2D receptors on immune cells, such as NK cells and cytotoxic T cells. Binding of NKG2DL to the NKG2D receptor on NK cells triggers their cytotoxic effects, resulting in NK cell-mediated lysis of target cells (22). As the number of NK cells returns to near-normal levels in the early post-transplant phase, the NK cell-mediated immunological effects thus contribute to the clearance of LSCs (23). The incorporation of olaparib into the conditioning regimen enhanced the eradication of LSCs by post-transplantation NK cells and cytotoxic T cells. This mechanism facilitated early achievement of CMR in RUNX1::RUNX1T1+ leukemia post-transplant, ultimately contributing to long-term disease control.

Although intensified conditioning regimens enhance transplant outcomes, relapse is still frequent after allo-HSCT for high risk RUNX1::RUNX1T1+ leukemia, even when carried out under CR1 conditions with intensified conditioning (24). For RUNX1::RUNX1T1-positive leukemia patients who fail to achieve MMR after allogeneic hematopoietic stem cell transplantation, the risk of relapse is significantly elevated. Appropriate maintenance therapy may help reduce this risk (25). Adding olaparib to our transplant conditioning regimen exploits the DNA repair defect in RUNX1::RUNX1T1+ leukemia cells, thus enhancing the effectiveness of cytotoxic agents in eliminating these cells during conditioning. Moreover, olaparib may harness underlying immune mechanisms to promote the clearance of LSCs. This combined effect probably accounts for the favorable treatment outcomes observed in our study. The introduction of olaparib has reduced the need for post-transplant maintenance therapy.

Despite being a retrospective study with a limited sample size, the inherent limitations may undermine the reliability of the conclusions. Our preliminary study indicates that incorporating olaparib into the conditioning regimen for leukemia transplantation is safe. Its unique mechanism of action may improve the outcomes of allo-HSCT in patients with relapsed and refractory RUNX1::RUNX1T1+ AML, rigorous clinical studies are necessary for further exploration and validation.

## Data Availability

The original contributions presented in the study are included in the article/supplementary material. Further inquiries can be directed to the corresponding author.
